# Supplementary material for: Environmental palaeogenomic reconstruction of an Ice Age algal population
Source: Commun Biol. 2021 Feb 16;4:220. doi: 10.1038/s42003-021-01710-4 (PMC7887274; doi:10.1038/s42003-021-01710-4)
Supplement: Supplementary file 2 — Supplementary Information [file 42003_2021_1710_MOESM2_ESM.pdf]

Environmental palaeogenomic reconstruction of an Ice Age algal population

Youri Lammers[1,#], Peter D. Heintzman[1,*], Inger Greve Alsos[1,*]

[1]The Arctic University Museum of Norway, UiT - The Arctic University of Norway, Tromsø, Norway

[*]Contributed equally to this work

[#]Corresponding author: e-mail: youri.lammers@uit.no

Supplementary Methods

Metabarcoding analysis of Lake Øvre Æråsvatnet material

153 *sed*aDNA extracts (see Main text for extraction method) from Lake Øvre Æråsvatnet[1] along with 78 controls, consisting out of 30 extraction negatives, 24 PCR negatives and 24 PCR positives were metabarcoded with the *trn*L g/h p6 loop primers[2]. Each metabarcoding PCR for a *sed*aDNA sample was replicated eight times, while the control PCR samples were replicated four times. Both the amplification and clean-up followed the protocol described by Alsos *et al.* 2016[3]. PCR products were converted into four Illumina amplicon libraries with the MetaFast method (FASTERIS SA, Switzerland) and the resulting libraries were sequenced on an Illumina HiSeq 2500 at 2 × 100 bp paired-end sequencing. The bioinformatic analysis and filtering steps followed that described by Alsos *et al.* 2020[1] (tag-sample lookup tables are provided in Supplementary Data 8).

Meta-analysis of *Nannochloropsis* in previous sedaDNA data sets

We performed a meta-analysis of the global prevalence of *Nannochloropsis* since the last ice age using published and available lake sedaDNA data sets. Three published shotgun datasets from Lake Hill, Alaska, USA[4,5], Charlie Lake, BC and Spring Lake, Alberta, Canada[6], and Hässeldala Port, Sweden[7] were reanalysed for the presence of *N. limnetica* using the same nuclear genome method used in this study (Supplementary Data 7). Furthermore, a metabarcode data set was reanalysed from Skartjørna, Svalbard[3], using the same methods for analysis as the original study, but lowering the minimum barcode length to 10 bp, in order to

retain the *Nannochloropsis* barcode (tag-sample lookup is provided in Supplementary Data 8). These data sets were supplemented with *sed*aDNA metabarcoding studies that reported *Nannochloropsis*, including; Bliss Lake, Greenland[8], Qinghai Lake, China[9], Lielais Svẽtiņu, Latvia[10], Lake Øvre Æråsvatnet[1], and Jodavannet, Svalbard[11].

We estimated the occurrence and abundance of *Nannochloropsis* in 5,000-year time windows for the above data sets. Abundance was coarsely divided into four categories for the metabarcode data: (1) dominant, scored when *Nannochloropsis* was the only taxon detected or most abundant of the taxa identified in the sequence data; (2) common, assigned when it was in the top 10 most abundant taxa identified; (3) rare, scored for any other detections, and (4) absent, assigned if *Nannochloropsis* was not detected. The reanalysed shotgun data sets were scored as: (1) dominant, when *Nannochloropsis* made up >=0.1% of the filtered read data; (2) common, 0.09-0.01%; (3) rare, 0.009-0.001%, and (4) absent, with <0.001%.

Supplementary Discussion

In total three *Nannochloropsis* p6-loop variants were detected in the metabarcode data, with the two most abundant variants matching the variants detected in the reconstructed palaeogenomes. The third variant was rarer and could only be detected in five samples. The detections of the two abundant *Nannochloropsis* p6-loop sequences are comparable to those reported by Alsos *et al.* 2020[1], where the algae was primarily detected in lithological units U2 and U3a (Supplementary Figure 11) and disappeared higher into the cores. The rarer variant was only detected in two samples for Alsos *et al.* 2020[1] and was not replicated in the same layers for this study (Supplementary Figure 11).

The two abundant *Nannochloropsis* p6-loop sequences were not found in the extraction blanks, or negative and positive PCR controls (Supplementary Figure 11), nor were they detected in the metabarcoding controls by Alsos *et al.* 2020[1]. The third barcode, which is identified as *N. granulata* (Supplementary Table 5) was observed in four controls (two extraction blanks, one PCR negative and one PCR positive control), as well as in one PCR positive control by Alsos *et al.* 2020[1] (Supplementary Figure 11). The occurrences of this *N. granulata* sequence is both rare and poorly replicated in the *sed*aDNA extracts and given the lack of co-occurrence with the two other *Nannochloropsis* sequences, is unlikely to be a PCR artefact or a related *Nannochloropsis* haplotype. Furthermore, the *N. granulata* barcode could not be observed in the mapped *Nannochloropsis* chloroplast shotgun sequence

data. This suggests that the *N. granulata* sequence has an independent origin compared to the two other detected *Nannochloropsis* metabarcode variants. Given the occurrence in some of the controls and the lack of replication in the *sed*aDNA samples, the *N. granulata* detection is not considered trustworthy, unlike the other two variants. This may have resulted from a taxonomic misassignment in the metabarcode reference databases or from haplotypic convergence between *Nannochloropsis* and an unknown contaminant, the latter of which we infer to be detecting here.

Supplementary References

1. Alsos, I. G. *et al.* Last Glacial Maximum environmental conditions at Andøya, northern Norway; evidence for a northern ice-edge ecological 'hotspot'. *Quat. Sci. Rev.* **239**, 106364 (2020).
2. Taberlet, P. *et al.* Power and limitations of the chloroplast trn L (UAA) intron for plant DNA barcoding. *Nucleic Acids Res.* **35**, e14–e14 (2007).
3. Alsos, I. G. *et al.* Sedimentary ancient DNA from Lake Skartjørna, Svalbard: Assessing the resilience of arctic flora to Holocene climate change. *Holocene* **26**, 627–642 (2016).
4. Graham, R. W. *et al.* Timing and causes of mid-Holocene mammoth extinction on St. Paul Island, Alaska. *Proc. Natl. Acad. Sci. U.S.A.* **113**, 9310–9314 (2016).
5. Wang, Y. *et al.* The southern coastal Beringian land bridge: cryptic refugium or pseudorefugium for woody plants during the Last Glacial Maximum? *J. Biogeogr.* **44**, 1559–1571 (2017).
6. Pedersen, M. W. *et al.* Postglacial viability and colonization in North America's ice-free corridor. *Nature* vol. 537 45–49 (2016).
7. Parducci, L. *et al.* Shotgun Environmental DNA, Pollen, and Macrofossil Analysis of Lateglacial Lake Sediments From Southern Sweden. *Front. Ecol. Evol.* **7**, (2019) doi:10.3389/fevo.2019.00189.
8. Epp, L. S. *et al.* Lake sediment multi-taxon DNA from North Greenland records early post-glacial appearance of vascular plants and accurately tracks environmental changes. *Quat. Sci. Rev.* **117**, 152–163 (2015).
9. Li, G. *et al.* Temporal Succession of Ancient Phytoplankton Community in Qinghai Lake and Implication for Paleo-environmental Change. *Sci. Rep.* **6**, 19769 (2016).
10. Stivrins, N. *et al.* Towards understanding the abundance of non-pollen palynomorphs: A

comparison of fossil algae, algal pigments and seda DNA from temperate lake sediments. *Rev. Palaeobot. Palynol.* **249**, 9–15 (2018).

11. Voldstad, L. H. *et al.* A complete Holocene lake sediment ancient DNA record reveals long-standing high Arctic plant diversity hotspot in northern Svalbard. *Quat. Sci. Rev.* **234**, 106207 (2020).
12. Alsos, I. G. *et al.* Plant DNA metabarcoding of lake sediments: How does it represent the contemporary vegetation. *PLoS One* **13**, e0195403 (2018).

Supplementary Figures

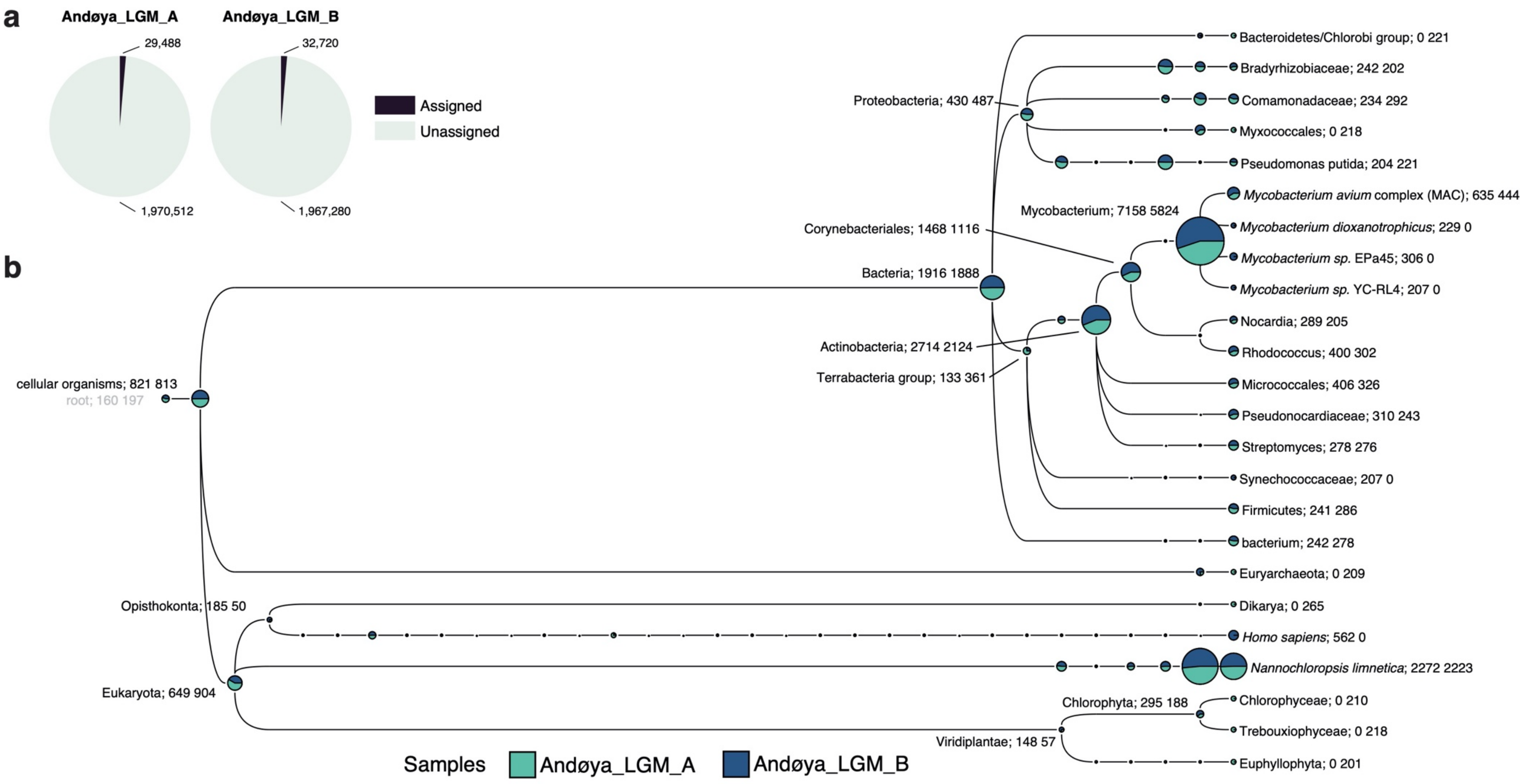

Supplementary Figure 1: Visualization of results from the BLAST-based metagenomic analysis.

(a) The proportion of assigned reads for each sample. (b) The metagenomic breakdown of the taxa present. All tips are collapsed to the genus-level or higher, with the exception of the two most read-abundant tips: *Mycobacterium* and *Nannochloropsis*. The numbers represent the reads assigned to each node for samples Andøya_LGM_B and Andøya_LGM_A respectively.

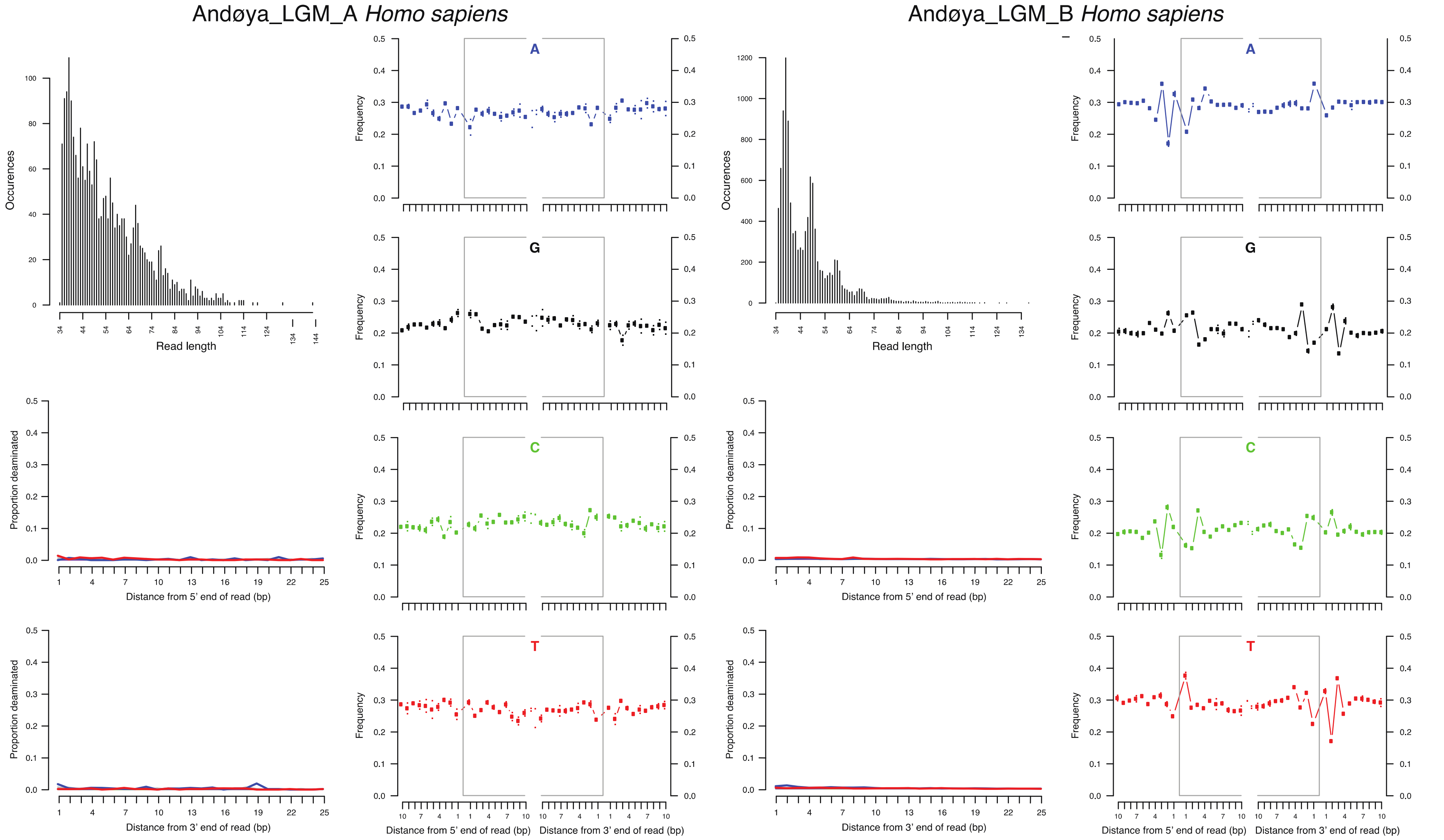

Supplementary Figure 2: Ancient DNA damage patterns for sequences aligned to the *Homo sapiens* nuclear reference genome as estimated by mapDamage.

In the deamination plot panels, red and blue lines respectively represent cytosine deamination profiles at the 5' and 3' end of aligned sequences.

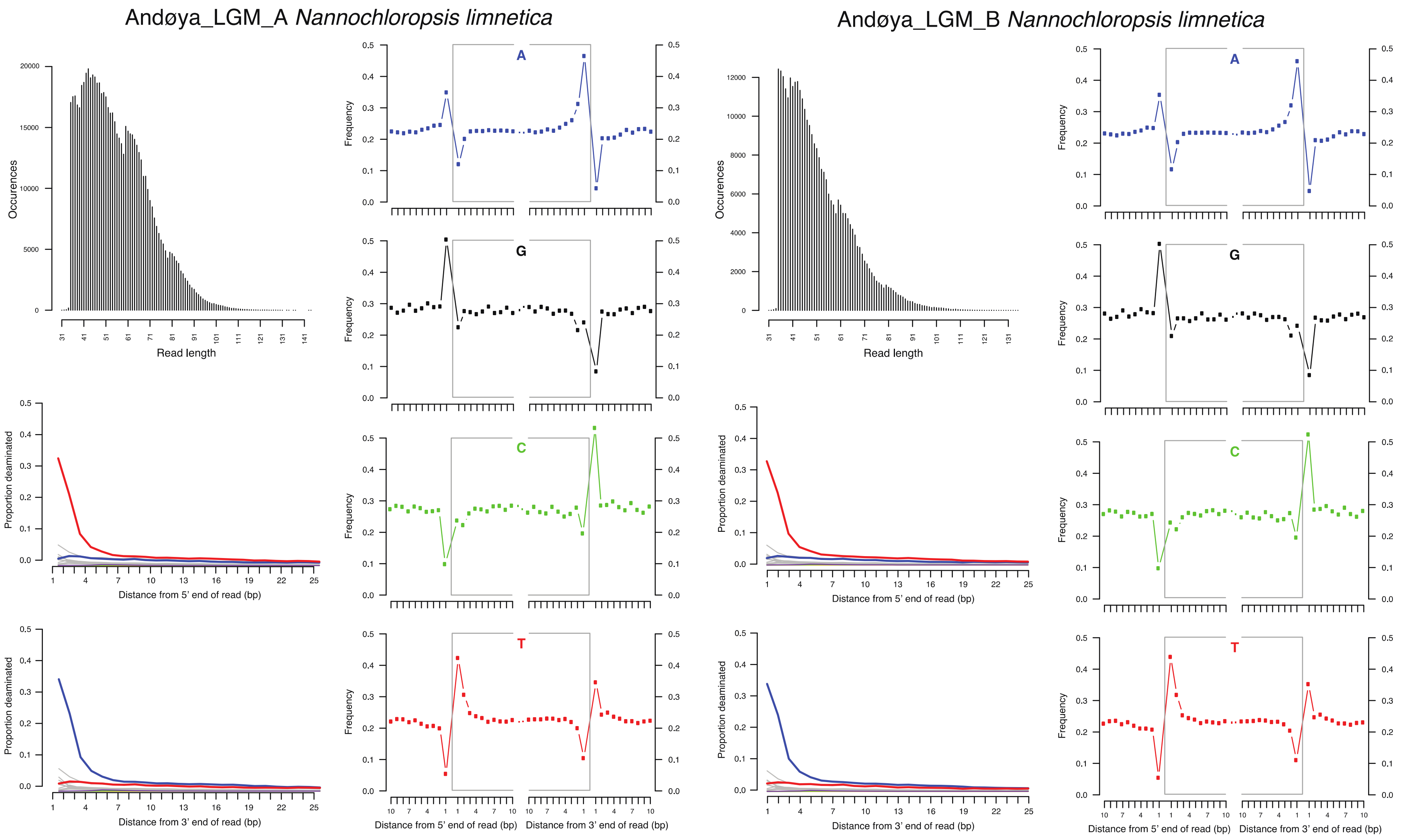

Supplementary Figure 3: Ancient DNA damage patterns for sequences aligned to the *Nannochloropsis limnetica* nuclear reference genome as estimated by mapDamage.

In the deamination plot panels, red and blue lines respectively represent cytosine deamination profiles at the 5' and 3' end of aligned sequences.

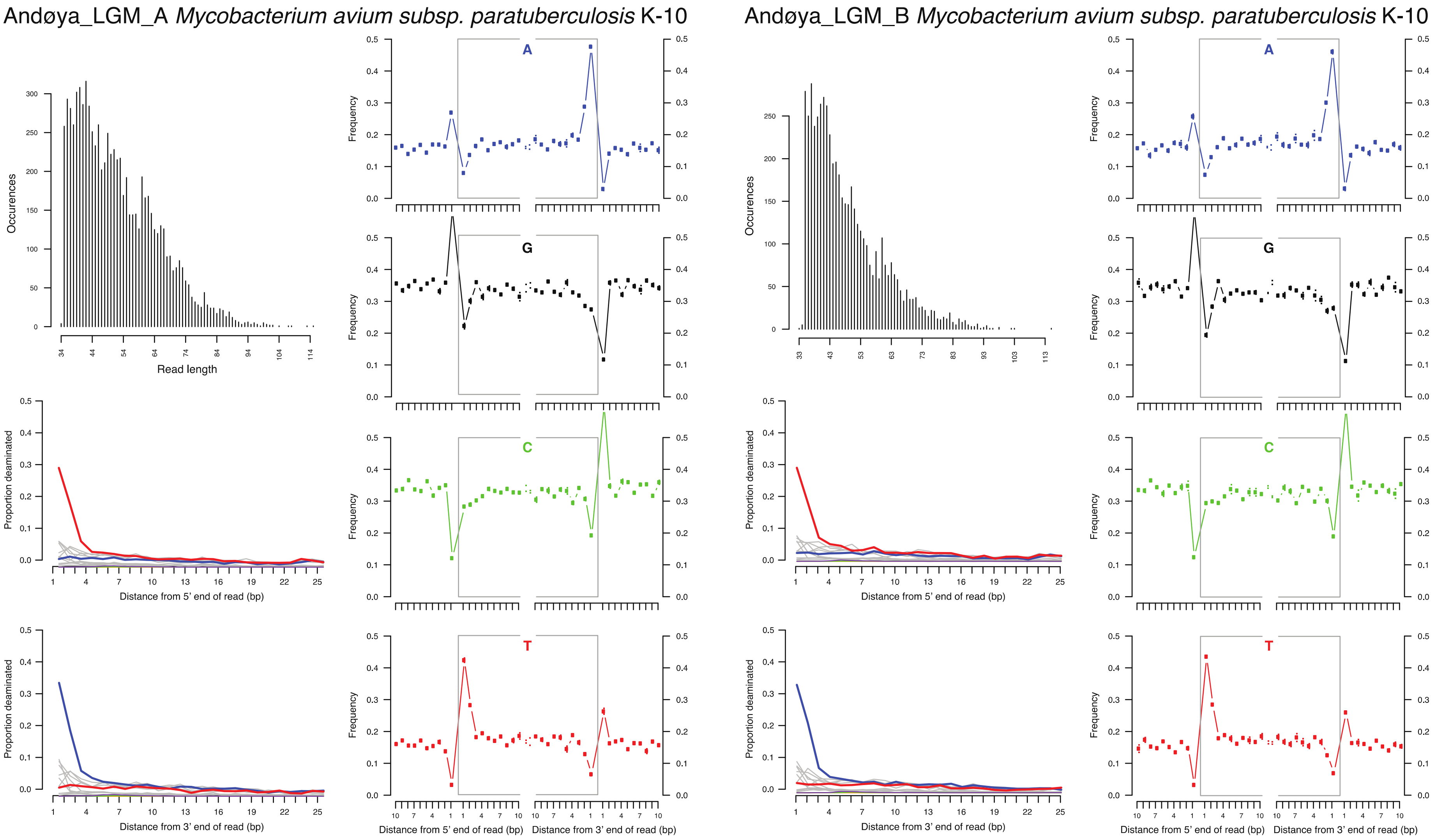

Supplementary Figure 4: Ancient DNA damage patterns for sequences aligned to the *Mycobacterium avium* nuclear reference genome as estimated by mapDamage.

In the deamination plot panels, red and blue lines respectively represent cytosine deamination profiles at the 5' and 3' end of aligned sequences.

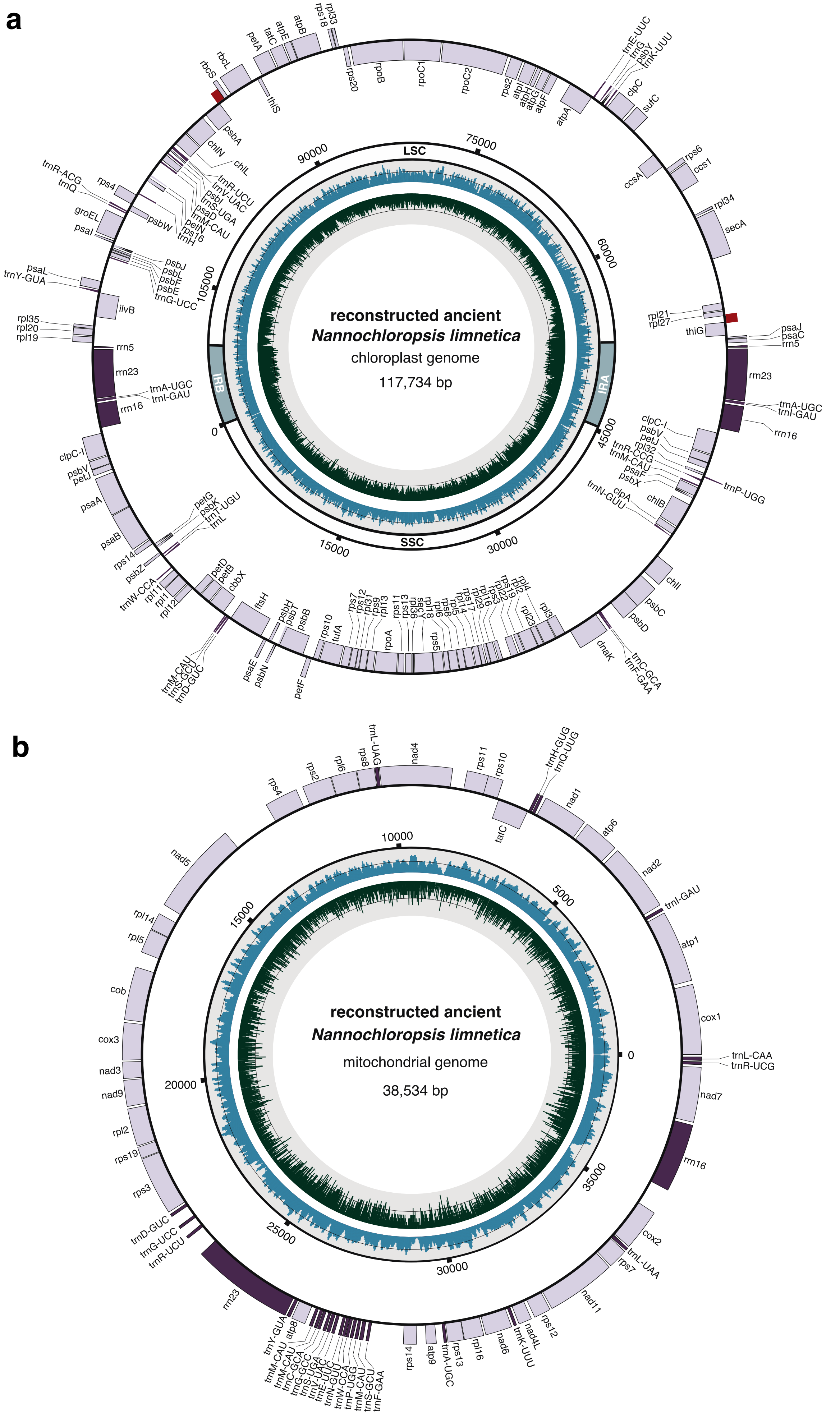
a
reconstructed ancient
Nannochloropsis limnetica
chloroplast genome
117,734 bp
LSC
SSC
IRA
IRB
b
reconstructed ancient
Nannochloropsis limnetica
mitochondrial genome
38,534 bp

Supplementary Figure 5: Fully annotated maps for the reconstructed *Nannochloropsis limnetica* organellar genomes.

Fully annotated maps for the reconstructed *N. limnetica* (a) chloroplast and (b) mitochondrial palaeogenomes. The innermost circle contains a distribution of the GC content in dark green, with the black line representing the 50% mark. The outer distribution contains the coverage for the assembly in blue, with the black line representing the average coverage of 64.3x for the chloroplast and 64.9x for the mitochondria. For the chloroplast the inverted repeats (IRA and IRB), large single copy (LSC) and small single copy (SSC) regions are annotated. The genomic features are given on the outermost circle, where the coding genes are coloured light purple and RNAs in dark purple. The features located on the inside are transcribed clockwise, those on the outside anticlockwise. The red bars on the outermost circle of the chloroplast indicate the location of the two regions with structural change compared to the *N. limnetica* reference genome.

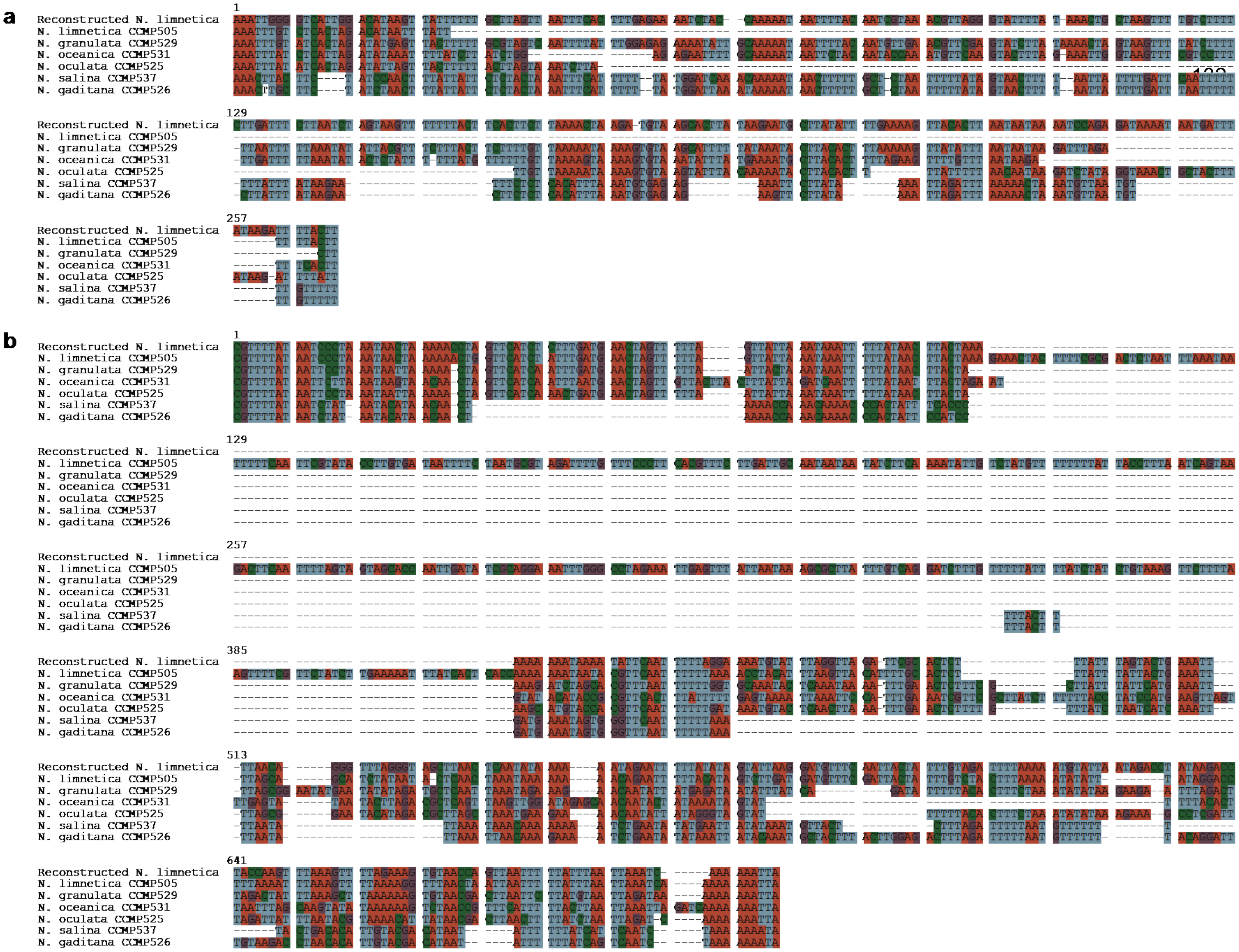
a
b
Reconstructed N. limnetica
N. limnetica CCMP505
N. granulata CCMP529
N. oceanica CCMP531
N. oculata CCMP525
N. salina CCMP537
N. gaditana CCMP526

Supplementary Figure 6: Alignments for the two major structural changes in the reconstructed chloroplast compared to the six reference *Nannochloropsis* chloroplast genomes.

(a) A 233 bp insertion in a non-coding region between the *thiG* and *rpl27* genes, which is absent in the reference *N. limnetica* chloroplast. (b) A 323 bp deletion compared to the reference *N. limnetica* chloroplast in a non-coding region between the *rbcS* and *psbA* genes. The accession codes for the *Nannochloropsis* sequences are provided in Supplementary Data 2.

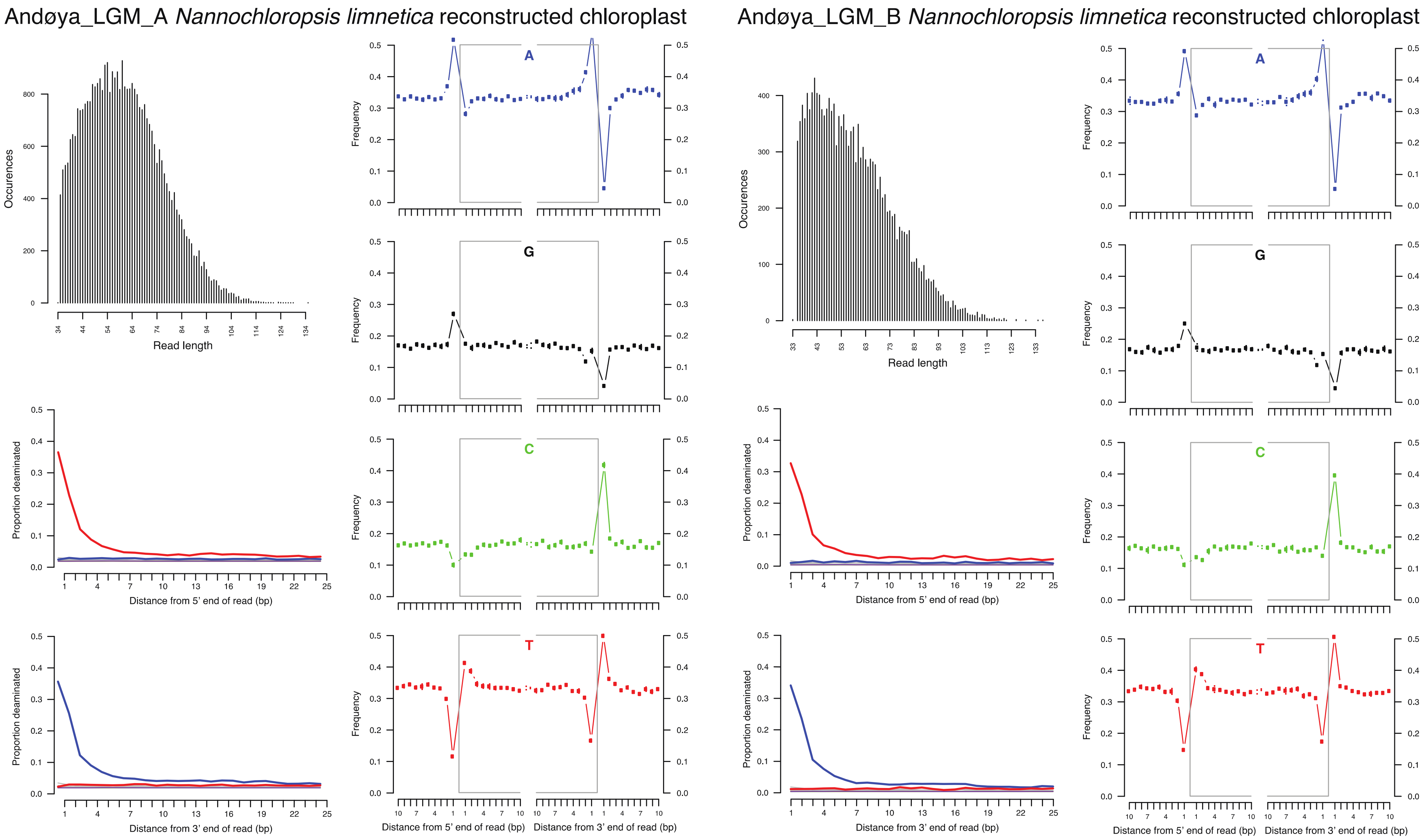

Supplementary Figure 7: Ancient DNA damage patterns for sequences aligned to the reconstructed *Nannochloropsis limnetica* chloroplast genome as estimated by mapDamage.

In the deamination plot panels, red and blue lines respectively represent cytosine deamination profiles at the 5’ and 3’ end of aligned sequences.

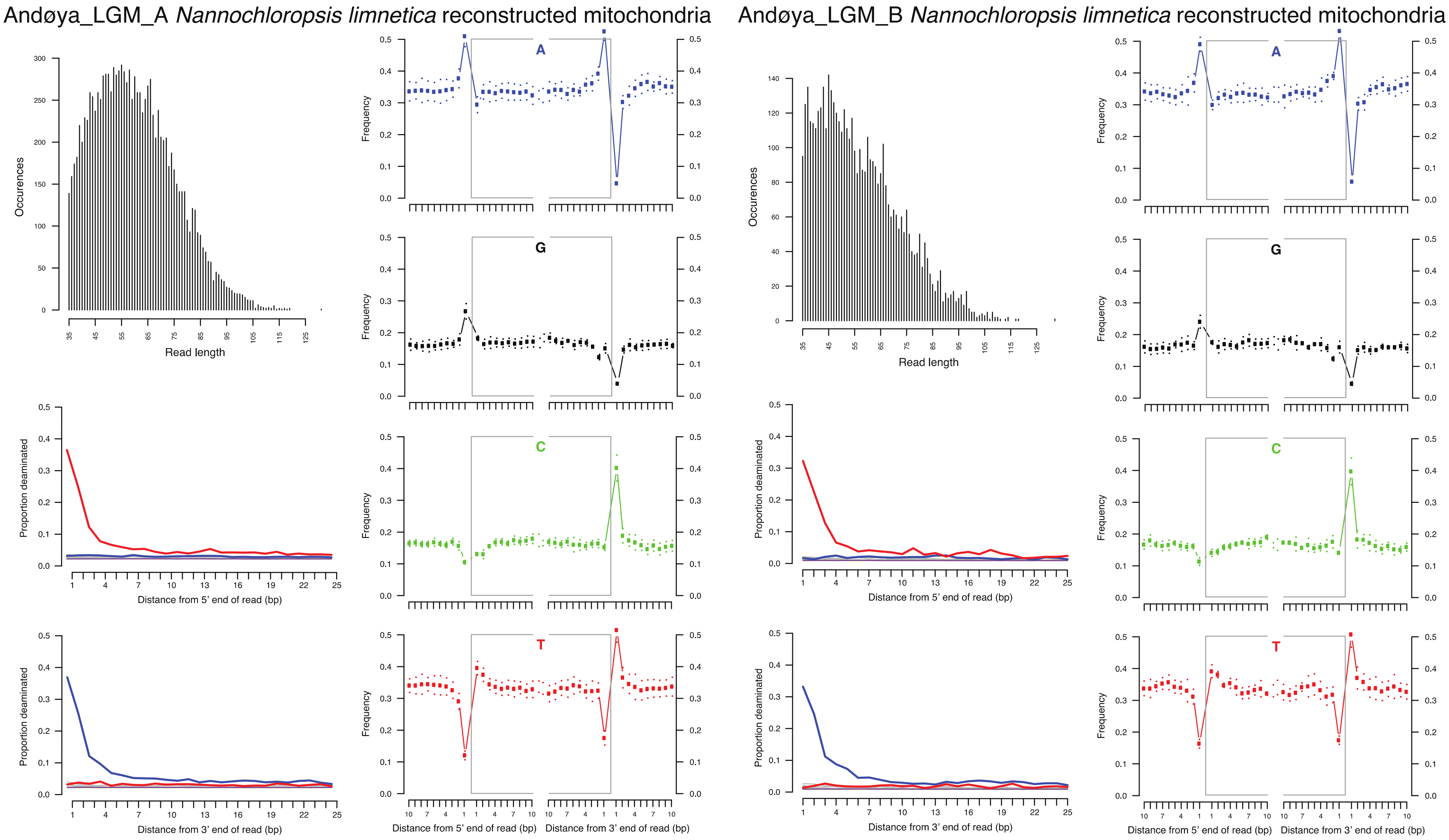

Supplementary Figure 8: Ancient DNA damage patterns for sequences aligned to the reconstructed *Nannochloropsis limnetica* mitochondrial genome as estimated by mapDamage.

In the deamination plot panels, red and blue lines respectively represent cytosine deamination profiles at the 5' and 3' end of aligned sequences.

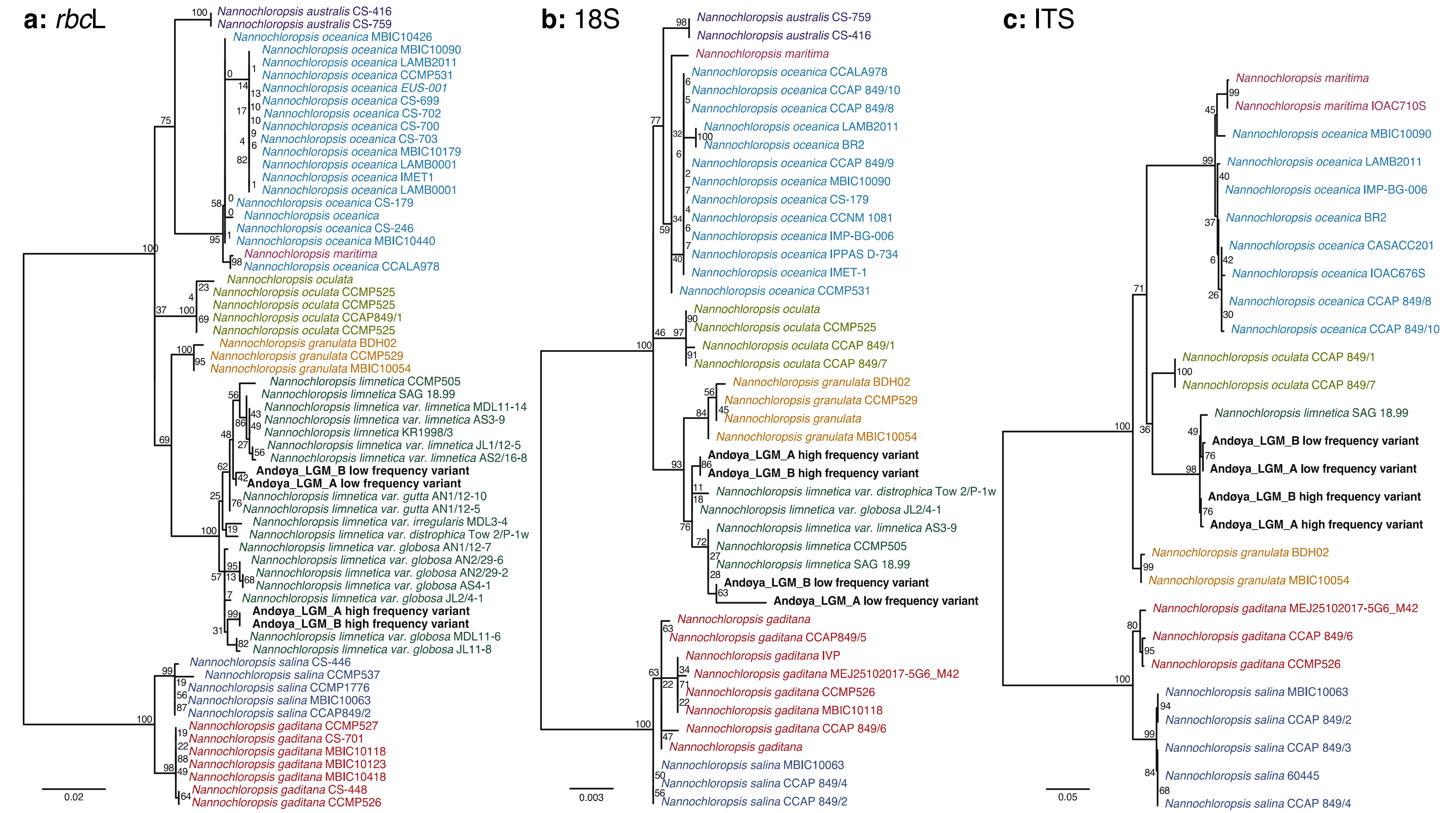
a: rbcL
Nannochloropsis australis CS-416
Nannochloropsis australis CS-759
Nannochloropsis oceanica MBIC10426
Nannochloropsis oceanica MBIC10090
Nannochloropsis oceanica LAMB2011
Nannochloropsis oceanica CCMP531
Nannochloropsis oceanica EUS-001
Nannochloropsis oceanica CS-699
Nannochloropsis oceanica CS-702
Nannochloropsis oceanica CS-700
Nannochloropsis oceanica CS-703
Nannochloropsis oceanica MBIC10179
Nannochloropsis oceanica LAMB0001
Nannochloropsis oceanica IMET1
Nannochloropsis oceanica LAMB0001
Nannochloropsis oceanica CS-179
Nannochloropsis oceanica
Nannochloropsis oceanica CS-246
Nannochloropsis oceanica MBIC10440
Nannochloropsis maritima
Nannochloropsis oceanica CCALA978
Nannochloropsis oculata
Nannochloropsis oculata CCMP525
Nannochloropsis oculata CCMP525
Nannochloropsis oculata CCAP849/1
Nannochloropsis oculata CCMP525
Nannochloropsis granulata BDH02
Nannochloropsis granulata CCMP529
Nannochloropsis granulata MBIC10054
Nannochloropsis limnetica CCMP505
Nannochloropsis limnetica SAG 18.99
Nannochloropsis limnetica var. limnetica MDL11-14
Nannochloropsis limnetica var. limnetica AS3-9
Nannochloropsis limnetica KR1998/3
Nannochloropsis limnetica var. limnetica JL1/12-5
Nannochloropsis limnetica var. limnetica AS2/16-8
Andøya_LGM_B low frequency variant
Andøya_LGM_A low frequency variant
Nannochloropsis limnetica var. gutta AN1/12-10
Nannochloropsis limnetica var. gutta AN1/12-5
Nannochloropsis limnetica var. irregularis MDL3-4
Nannochloropsis limnetica var. distrophica Tow 2/P-1w
Nannochloropsis limnetica var. globosa AN1/12-7
Nannochloropsis limnetica var. globosa AN2/29-6
Nannochloropsis limnetica var. globosa AN2/29-2
Nannochloropsis limnetica var. globosa AS4-1
Nannochloropsis limnetica var. globosa JL2/4-1
Andøya_LGM_A high frequency variant
Andøya_LGM_B high frequency variant
Nannochloropsis limnetica var. globosa MDL11-6
Nannochloropsis limnetica var. globosa JL11-8
Nannochloropsis salina CS-446
Nannochloropsis salina CCMP537
Nannochloropsis salina CCMP1776
Nannochloropsis salina MBIC10063
Nannochloropsis salina CCAP849/2
Nannochloropsis gaditana CCMP527
Nannochloropsis gaditana CS-701
Nannochloropsis gaditana MBIC10118
Nannochloropsis gaditana MBIC10123
Nannochloropsis gaditana MBIC10418
Nannochloropsis gaditana CS-448
Nannochloropsis gaditana CCMP526
0.02
b: 18S
Nannochloropsis australis CS-759
Nannochloropsis australis CS-416
Nannochloropsis maritima
Nannochloropsis oceanica CCALA978
Nannochloropsis oceanica CCAP 849/10
Nannochloropsis oceanica CCAP 849/8
Nannochloropsis oceanica LAMB2011
Nannochloropsis oceanica BR2
Nannochloropsis oceanica CCAP 849/9
Nannochloropsis oceanica MBIC10090
Nannochloropsis oceanica CS-179
Nannochloropsis oceanica CCNM 1081
Nannochloropsis oceanica IMP-BG-006
Nannochloropsis oceanica IPPAS D-734
Nannochloropsis oceanica IMET-1
Nannochloropsis oceanica CCMP531
Nannochloropsis oculata
Nannochloropsis oculata CCMP525
Nannochloropsis oculata CCAP 849/1
Nannochloropsis oculata CCAP 849/7
Nannochloropsis granulata BDH02
Nannochloropsis granulata CCMP529
Nannochloropsis granulata
Nannochloropsis granulata MBIC10054
Andøya_LGM_A high frequency variant
Andøya_LGM_B high frequency variant
Nannochloropsis limnetica var. distrophica Tow 2/P-1w
Nannochloropsis limnetica var. globosa JL2/4-1
Nannochloropsis limnetica var. limnetica AS3-9
Nannochloropsis limnetica CCMP505
Nannochloropsis limnetica SAG 18.99
Andøya_LGM_B low frequency variant
Andøya_LGM_A low frequency variant
Nannochloropsis gaditana
Nannochloropsis gaditana CCAP849/5
Nannochloropsis gaditana IVP
Nannochloropsis gaditana MEJ25102017-5G6_M42
Nannochloropsis gaditana CCMP526
Nannochloropsis gaditana MBIC10118
Nannochloropsis gaditana CCAP 849/6
Nannochloropsis gaditana
Nannochloropsis salina MBIC10063
Nannochloropsis salina CCAP 849/4
Nannochloropsis salina CCAP 849/2
0.003
c: ITS
Nannochloropsis maritima
Nannochloropsis maritima IOAC710S
Nannochloropsis oceanica MBIC10090
Nannochloropsis oceanica LAMB2011
Nannochloropsis oceanica IMP-BG-006
Nannochloropsis oceanica BR2
Nannochloropsis oceanica CASACC201
Nannochloropsis oceanica IOAC676S
Nannochloropsis oceanica CCAP 849/8
Nannochloropsis oceanica CCAP 849/10
Nannochloropsis oculata CCAP 849/1
Nannochloropsis oculata CCAP 849/7
Nannochloropsis limnetica SAG 18.99
Andøya_LGM_B low frequency variant
Andøya_LGM_A low frequency variant
Andøya_LGM_B high frequency variant
Andøya_LGM_A high frequency variant
Nannochloropsis granulata BDH02
Nannochloropsis granulata MBIC10054
Nannochloropsis gaditana MEJ25102017-5G6_M42
Nannochloropsis gaditana CCAP 849/6
Nannochloropsis gaditana CCMP526
Nannochloropsis salina MBIC10063
Nannochloropsis salina CCAP 849/2
Nannochloropsis salina CCAP 849/3
Nannochloropsis salina 60445
Nannochloropsis salina CCAP 849/4
0.05

Supplementary Figure 9: Maximum likelihood phylogenies of *Nannochloropsis* including the reconstructed *N. limnetica* high and low frequency variant consensus sequences

Based on (a) ~1100 bp of the *rbc*L chloroplast locus, (b) ~1800 bp of the 18S nuclear locus, and (c) ~860 bp of the ITS nuclear locus. Accession codes and coordinates for the sequences are provided in Supplementary Data 4.

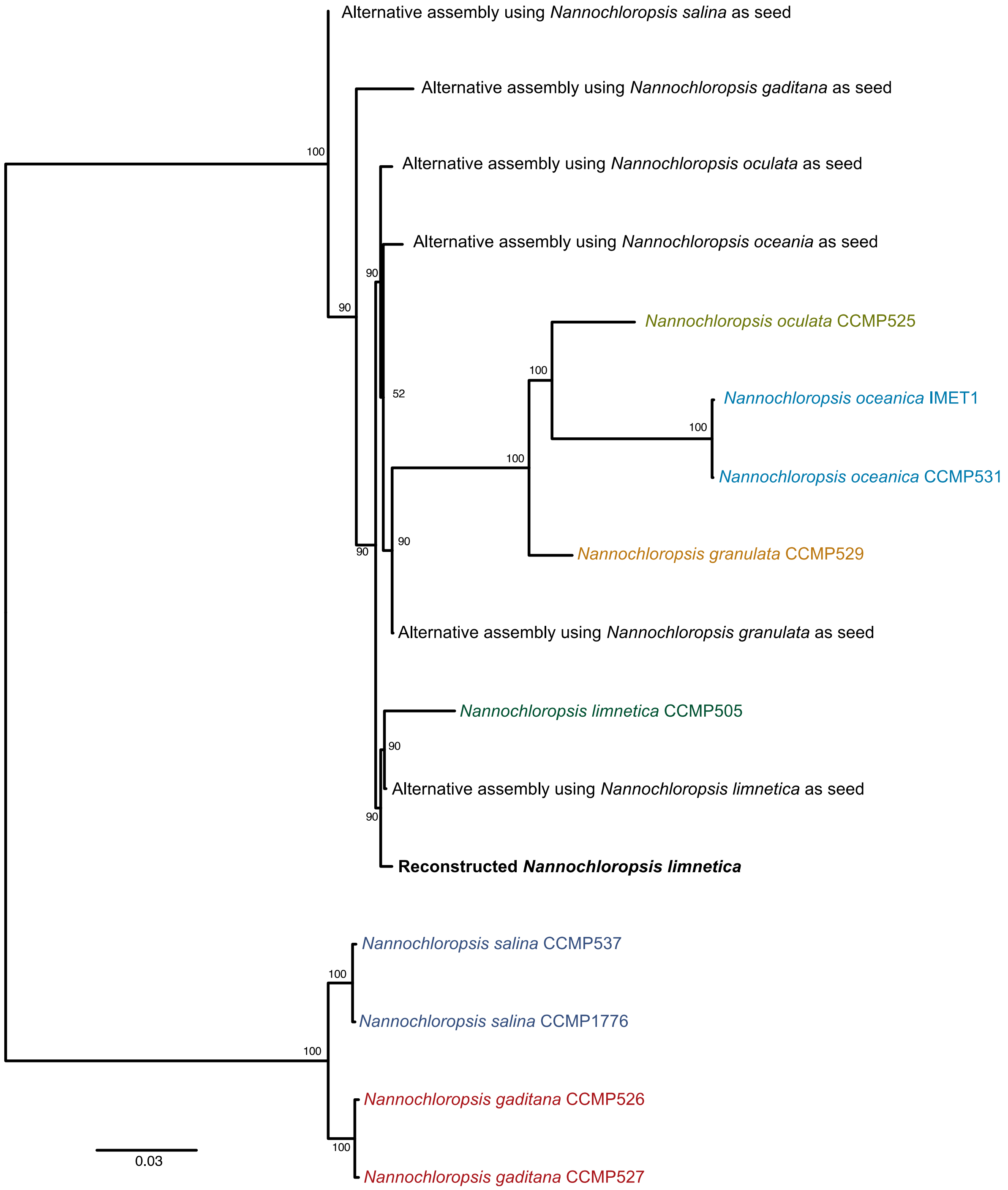

152 **Supplementary Figure 10: Maximum likelihood phylogeny of *Nannochloropsis***
153 **chloroplast genome sequences, including the reconstructed *N. limnetica* palaeogenomes**
154 **and the organellar genomes that used alternative *Nannochloropsis* taxa as seed**
155 **sequences.**

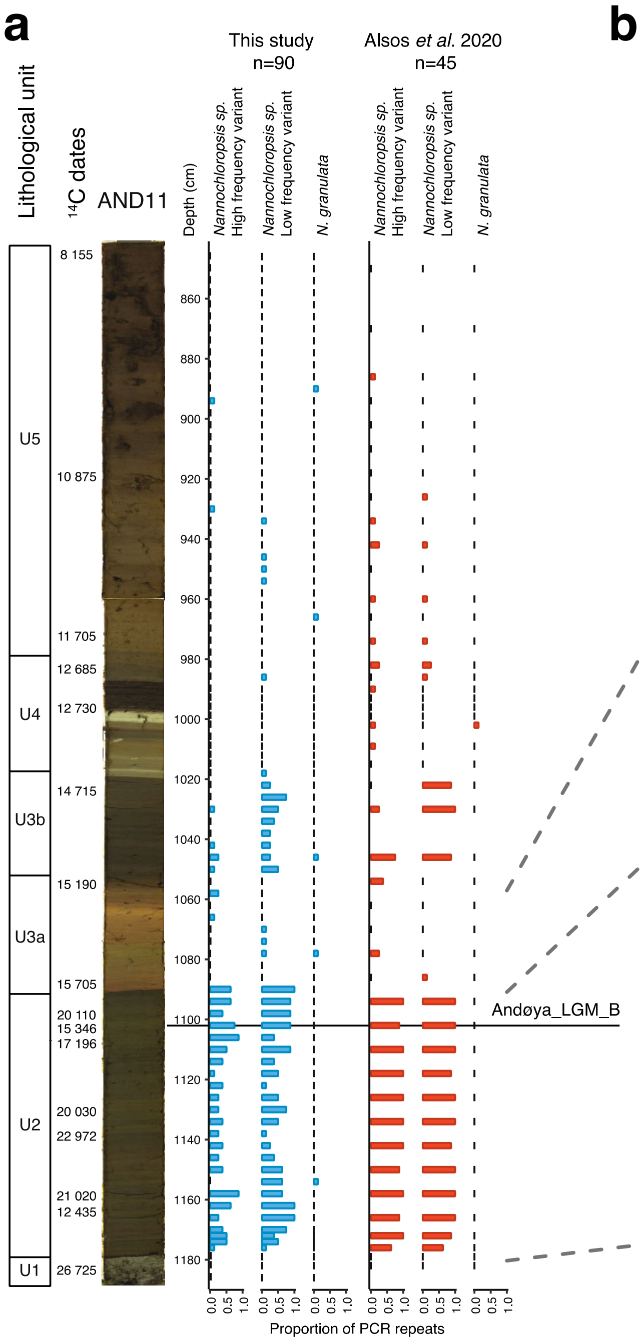

b

Study	Dataset	Number of samples	***Nannochloropsis* sp. High frequency variant**	***Nannochloropsis* sp. Low frequency variant**	***N. Granulata***	Total
This study	Lake *seda*DNA samples	153 (1224)	70 (226)	83 (295)	5 (5)	93 (526)
	Extraction negative controls	30 (120)	0	0	2 (4)	2 (4)
	PCR negative controls	24 (96)	0	0	1 (1)	1 (1)
	PCR positive controls	24 (96)	0	0	1 (1)	1 (1)
Alsos *et al.* 2020	Lake *seda*DNA samples	76 (608)	55 (302)	52 (309)	2 (4)	59 (615)
	Extraction negative controls	6 (48)	0	0	0	0
	PCR negative controls	6 (48)	0	0	0	0
	PCR positive controls	2 (16)	0	0	1 (1)	1 (1)

The number of samples are given with the number of PCR replicates in parentheses

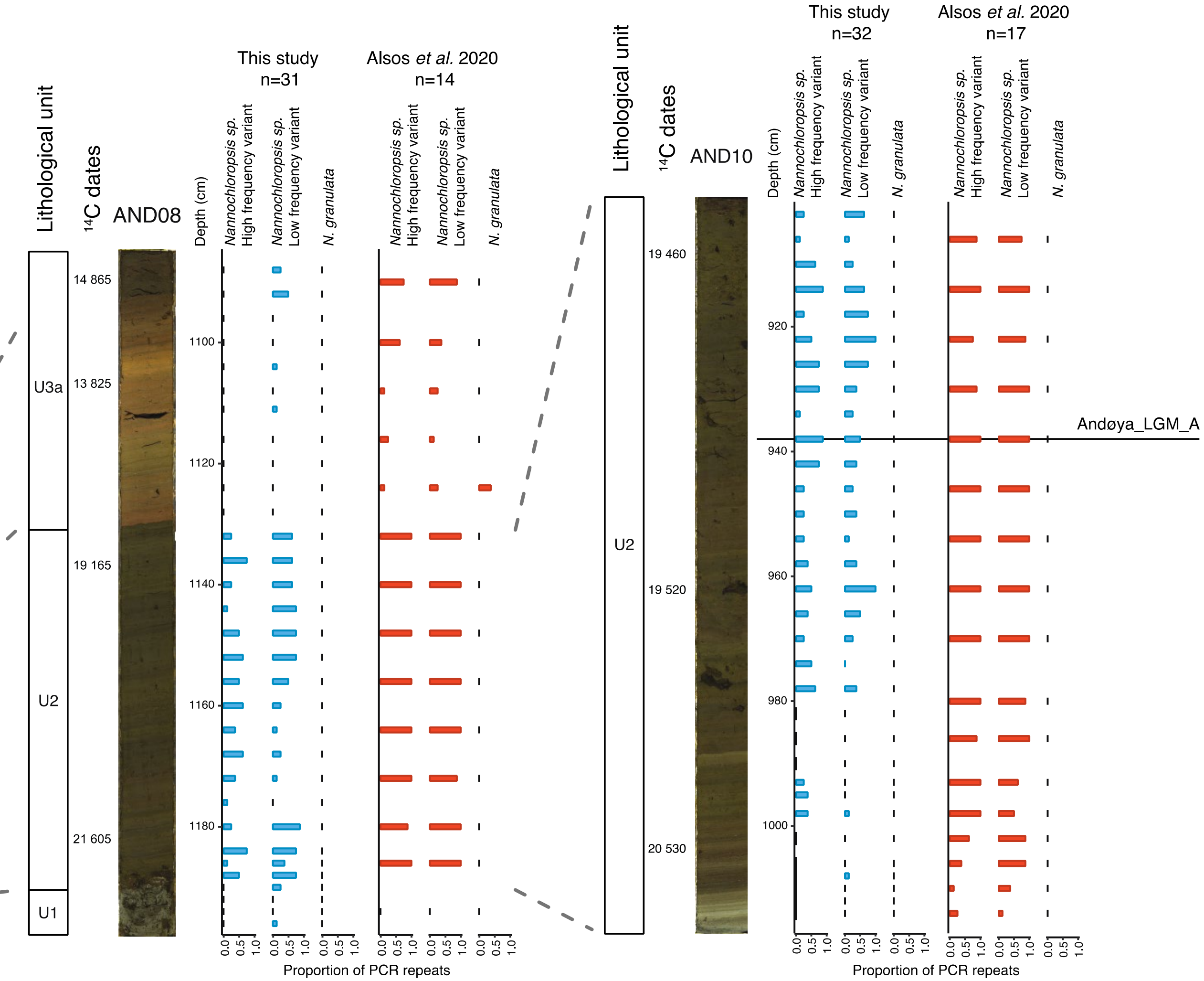

Supplementary Figure 11: Detection of *Nannochloropsis* in metabarcoding data sets from Lake Øvre Æråsvatnet. (a) Detection of *Nannochloropsis* in the Lake Øvre Æråsvatnet, Andøya, Norway cores based on the metabarcode data presented in this study and that of Alsos *et al.* 2020[1]. The metabarcode data, lithological units, and loss-on-ignition curves are visualized along the core images. For each *Nannochloropsis* p6-loop barcode, the proportion of PCR replicates that contain the variant is displayed. All detections are coloured blue or red for this study or Alsos *et al.* 2020[1] respectively, while non-detections are black. The two shotgun metagenomic samples; Andøya_LGM_A and Andøya_LGM_B are marked. Figure is adapted from Alsos *et al.* 2020[1]. (b) Summary of the *Nannochloropsis* metabarcoding detections in the Lake Øvre Æråsvatnet *sed*aDNA extracts and controls. The number of samples are given with the number of PCR replicates in parentheses.

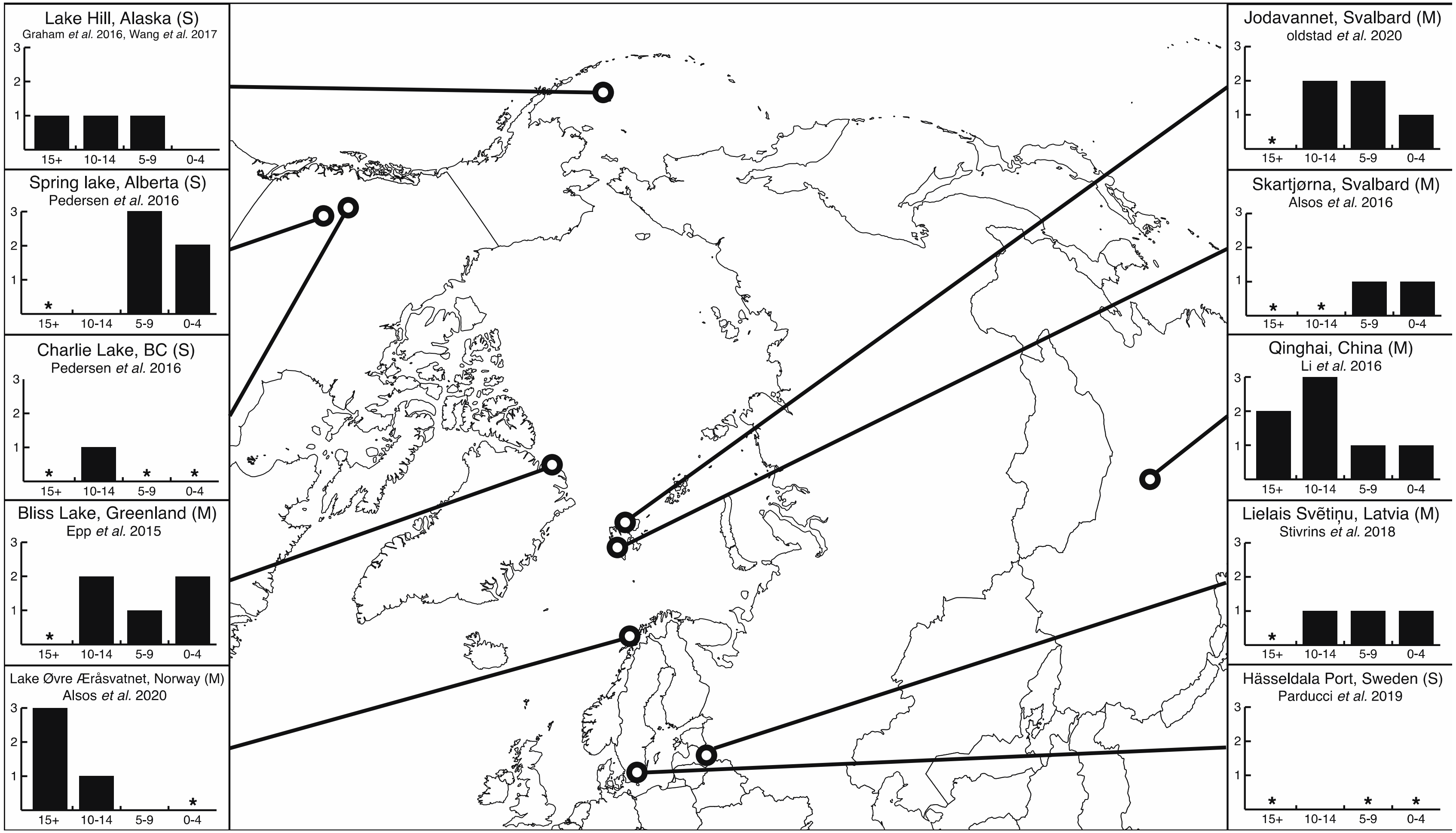

Supplementary Figure 12: Overview of *Nannochloropsis* detections, using 10 *sed*aDNA data sets either as previously published or based on reanalysis of available data.

Plotted on the Y-axis is the inferred abundance of *Nannochloropsis*; 3: dominant in the period, 2: common, 1: rare and 0 is absent. Data were binned into 5000 year time periods, which might obscure finer patterns. No *sed*aDNA data was available for time periods marked with an asterisk. Studies used either shotgun metagenomics (S) or metabarcoding (M) for *Nannochloropsis* detection. The map is made using data from Natural Earth.

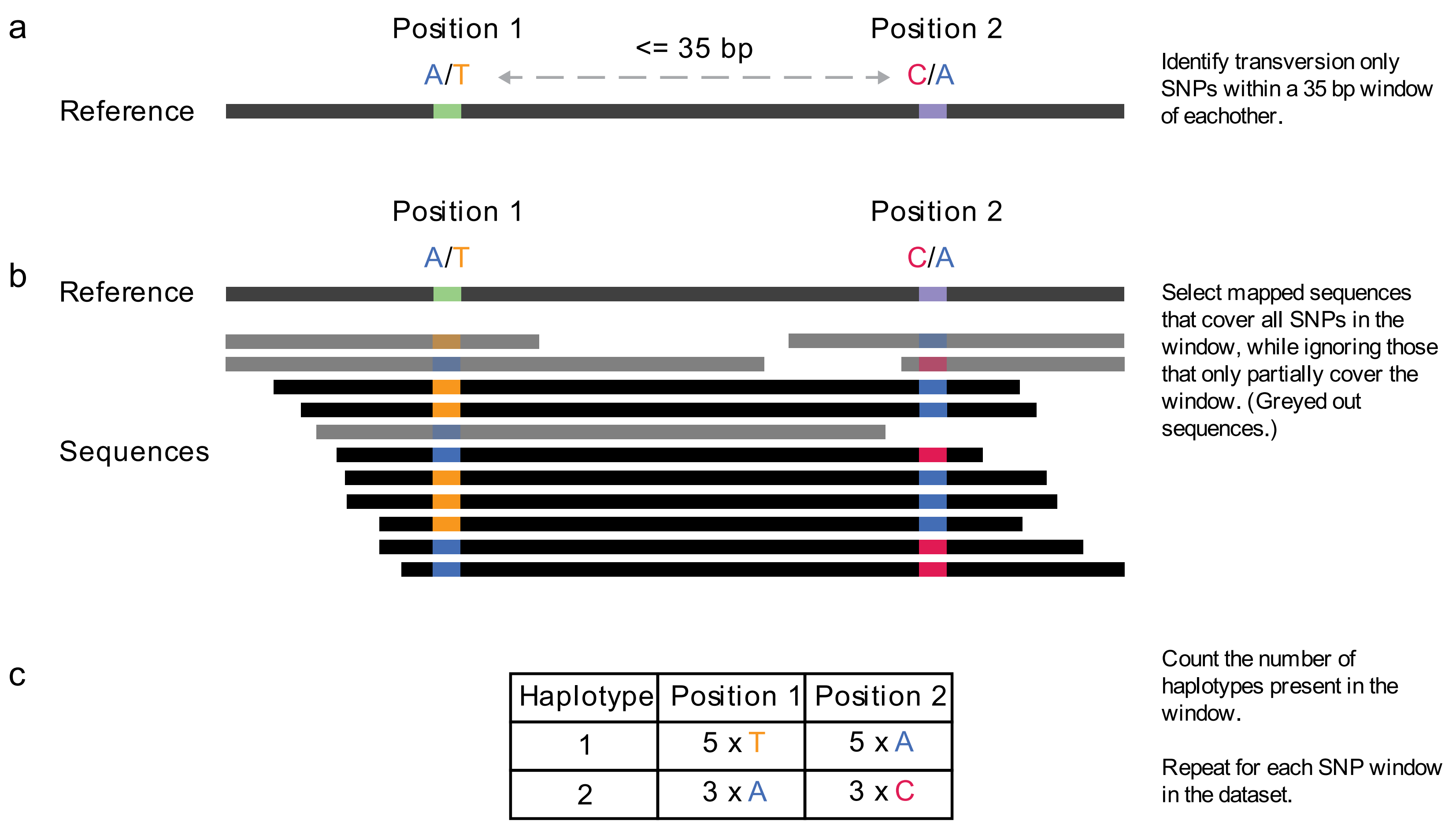

Supplementary Figure 13: Workflow of the haplotype calling method.

(a) Variant window selection. (b) Retrieval of candidate reads. (c) Scoring of the haplotypes present in the window.

Supplementary Tables

Supplementary Table 1: Summary statistics for the raw and filtered sequence read counts.

	Andøya_LGM_A	Andøya_LGM_B
Raw paired-end reads	223999748	133265048
SeqPrep: Merged reads with length ≥30 bp	171835490	75763460
SeqPrep: Percentage merged	76.71	56.85
SeqPrep: Discarded merged reads with length <30 bp	26994683	22731999
SeqPrep: Percentage discarded with length <30 bp	12.05	17.06
SeqPrep: Remaining unmerged reads	25169575	34769589
SeqPrep: Percentage unmerged	11.24	26.09
SeqPrep: Average merged read length	47.9	42.7
SGA: Merged reads with length >=35 bp	146920201	59188910
SGA: Percentage of raw reads, merged with length >= 35 bp	65.59	44.41
SGA: Average merged read length for reads >=35 bp	51.44	47.9
SGA: Final filtered reads (Max dust score of 1, >=35 bp)	127429489	53000389
SGA: Percentage of raw reads, post filtering	56.89	39.77
SGA: Average post-filtered read length	52.6	49.74

Supplementary Table 2: Summary statistics of the reconstructed N. limnetica organellar genomes, as well as the features on the N. limnetica reference.

	Reconstructed chloroplast	Reference chloroplast (NC_022262.1)	Reconstructed mitochondria	Reference mitochondria (NC_022256.1)
Length	117734	117806	38534	38543
Coverage based on the merged Andøya data	64.3x	60.8x	64.9x	62.4x
GC content	33.34	33.54	31.68	31.69
Coding DNA sequences	125	126	35	35
Transfer and ribosomal RNAs	34	34	28	28

Supplementary Table 3: Nannochloropsis chloroplast assemblies based on alternative reference sequences as seed. The reconstructed length, number of gaps, and the gap length is given for the alternative reconstructed chloroplasts.

Accession	Reference	Sequence length	Total gap length	Number of gaps	Ungapped length	% Ungapped
KJ410682.1	*Nannochloropsis gaditana* strain CCMP526 chloroplast, complete genome	114710	29499	1005	85211	74.28
KC598085.1	*Nannochloropsis granulata* chloroplast, complete genome	117672	3875	1615	113797	96.7
NC_022262.1	*Nannochloropsis limnetica* strain CCMP505 chloroplast, complete genome	117806	2159	1695	115647	98.17
NC_022263.1	*Nannochloropsis oceanica* strain CCMP531 chloroplast, complete genome	117556	5220	1548	112336	95.56
KC598087.1	*Nannochloropsis oculata* strain CCMP525 chloroplast, complete genome	117462	2654	1575	114808	97.74
NC_022261.1	*Nannochloropsis salina* strain CCMP537 chloroplast, complete genome	114735	17395	1266	97340	84.84

Supplementary Table 4: Variant ratios for each reconstructed Nannochloropsis organellar genome. The average number of transversion-only variants for each sample and organellar genome, as well as the average proportion of alternative alleles and the proportion retrieved in the damaged only datasets.

Sample	Reference	Average number of variants	SD number of variants	Average Variant Ratio	SD Variant Ratio	% Overlap with damaged only reads	SD Overlap damage only reads
Andøya_LGM_A	chloroplast	376.4	4.1593269	0.393	0.116	70.05	1.214
Andøya_LGM_B	chloroplast	299.2	2.2803509	0.419	0.122	64.052	0.8
Andøya_LGM_A	mitochondria	111.8	0.4472136	0.385	0.108	65.524	1.952
Andøya_LGM_B	mitochondria	80.6	0.8944272	0.427	0.108	67.948	2.027

Supplementary Table 5: Comparison of *Nannochloropsis* p6-loop reference sequences.

The reconstructed variant sequences are indicated in bold.

p6 loop sequence	Assignment	References
CTCACAAAAGTG	*Nannochloropsis salina*	*Nannochloropsis salina* (KJ410685)
CTCATAAAAATG	*Nannochloropsis sp.*	*Nannochloropsis gaditana* (KC012944, KC598084, KJ410682)<br>*N. limnetica* **(low frequency shotgun variant)**
CTCATAAAAGTG	*Nannochloropsis salina*	*Nannochloropsis salina* (KC598088)
CTCATGAAAATG	*Nannochloropsis sp.*	*Nannochloropsis oceanica* (CP038136, CP044582, CP044614, KC598086, KC598090, KJ410683)<br>*N. oculata* (KC598087, KJ410684)<br>*N. limnetica* (KC598089, **high frequency shotgun variant**)
CTCATGAAAGTG	*Nannochloropsis granulata*	*Nannochloropsis granulata* (KC598085)

Supplementary Table 6: Contemporary *Nannochloropsis* occurrences in northern Norway.

Detections of *Nannochloropsis* in a contemporary northern Norway environmental DNA metabarcoding data set[12].

Sample	Lake	District	Habitat type	Core	Depth	*Nannochloropsis* reads	*Nannochloropsis* PCR replicates (out of 6)
TTE01	Finnvatnet	Kvaløya	Birch forest/mire	Core1	0-2cm	0	0
TTE03	Lakselvhøgda	Ringvassøya	Alpine heath and mire	Core1	0-2cm	0	0
TTE04	Lakselvhøgda	Ringvassøya	Alpine heath and mire	Core1	2-4cm	0	0
TTE05	Lakselvhøgda	Ringvassøya	Alpine heath and mire	Core2	0-2cm	0	0
TTE06	Jula Jávri	Kåfjorddalen	Alpine heath and mire	Core1	0-2cm	736	5
TTE07	Jula Jávri	Kåfjorddalen	Alpine heath and mire	Core2	0-2cm	107	4
TTE09	Lauvås	Ringvassøya	Heath, mire and birch forest	Core1	0-2cm	0	0
TTE10	Lauvås	Ringvassøya	Heath, mire and birch forest	Core1	2-4cm	0	0
TTE11	Lauvås	Ringvassøya	Heath, mire and birch forest	Core2	0-2cm	0	0
TTE12	Øvre Æråsvatnet	Andøya	Mires and birch forest, conifers planted	Core1	0-2cm	0	0
TTE13	Øvre Æråsvatnet	Andøya	Mires and birch forest, conifers planted	Core2	0-2cm	0	0
TTE14	Paulan Jávri	Kåfjorddalen	Alpine heath	Core1	0-2cm	0	0
TTE15	Paulan Jávri	Kåfjorddalen	Alpine heath	Core2	0-2cm	0	0
TTE17	Gauptjern	Dividalen	Sub-alpine birch forest	Core1	0-2cm	0	0
TTE18	Gauptjern	Dividalen	Sub-alpine birch forest	Core1	2-4cm	26	1
TTE19	Gauptjern	Dividalen	Sub-alpine birch forest	Core1	4-6cm	0	0
TTE20	Gauptjern	Dividalen	Sub-alpine birch forest	Core2	0-2cm	0	0
TTE21	Gauptjern	Dividalen	Sub-alpine birch forest	Core2	2-4cm	0	0
TTE22	Gauptjern	Dividalen	Sub-alpine birch forest	Core2	4-6cm	0	0
TTE23	Brennskogtjønna	Dividalen	Pine forest	Core1	0-2cm	86	2
TTE25	Brennskogtjønna	Dividalen	Pine forest	Core1	2-4cm	0	0
TTE26	Brennskogtjønna	Dividalen	Pine forest	Core1	4-6cm	217	4
TTE27	Brennskogtjønna	Dividalen	Pine forest	Core2	0-2cm	358	3
TTE28	Brennskogtjønna	Dividalen	Pine forest	Core2	2-4cm	127	1
TTE29	Brennskogtjønna	Dividalen	Pine forest	Core2	4-6cm	179	1
TTE30	A-tjern	Dividalen	Birch forest/mire	Core1	0-2cm	3732	6
TTE31	A-tjern	Dividalen	Birch forest/mire	Core1	2-4cm	4317	6
TTE33	A-tjern	Dividalen	Birch forest/mire	Core1	4-6cm	4073	6
TTE34	A-tjern	Dividalen	Birch forest/mire	Core2	0-2cm	4528	6
TTE35	A-tjern	Dividalen	Birch forest/mire	Core2	2-4cm	7314	6
TTE36	A-tjern	Dividalen	Birch forest/mire	Core2	4-6cm	12899	6
TTE37	Rottjern	Dividalen	Pine and birch forest	Core1	0-2cm	5259	6
TTE38	Rottjern	Dividalen	Pine and birch forest	Core1	2-4cm	6092	6
TTE39	Rottjern	Dividalen	Pine and birch forest	Core1	4-6cm	4435	6
TTE41	Rottjern	Dividalen	Pine and birch forest	Core1	6-8cm	2273	6
TTE42	Rottjern	Dividalen	Pine and birch forest	Core2	0-2cm	2973	6
TTE43	Rottjern	Dividalen	Pine and birch forest	Core2	2-4cm	4170	6
TTE44	Rottjern	Dividalen	Pine and birch forest	Core2	4-6cm	2130	6
TTE45	Rottjern	Dividalen	Pine and birch forest	Core2	6-8cm	3565	6
TTE46	Einletvatnet	Andøya	Mires, patches of birch forest	Core1	0-2cm	0	0
TTE47	Einletvatnet	Andøya	Mires, patches of birch forest	Core2	0-2cm	0	0

Supplementary Table 7: Accession codes used for the annotation of the reconstructed organellar genomes.

Species	Chloroplast accession	Mitochondrial accession
Nannochloropsis gaditana	NC_020014.1	NC_020015.1
Nannochloropsis granulata	NC_022259.1	NC_022254.1
Nannochloropsis limnetica	NC_022262.1	NC_022256.1
Nannochloropsis oceanica	NC_022263.1	NC_022258.1
Nannochloropsis oculata	NC_022260.1	NC_022257.1
