# Supplementary material for: Environmental palaeogenomic reconstruction of an Ice Age algal population
Source: Commun Biol. 2021 Feb 16;4:220. doi: 10.1038/s42003-021-01710-4 (PMC7887274; doi:10.1038/s42003-021-01710-4)
Supplement: Supplementary file 3 — Description of Additional Supplementary Files [file 42003_2021_1710_MOESM3_ESM.pdf]

Description of Additional Supplementary Files

File name: Supplementary Data 1

Description: Tabulated MEGAN output for both samples and the non-overlapping one million read subsets.

File name: Supplementary Data 2

Description: The reference genomes used for the mapping analysis along with the raw and filtered sequence counts, corrected sequence counts and coverage for each sample. In addition, the shared and unique sequence counts are provided between *Nannochloropsis* nuclear and chloroplast genomes, as well as the *Mycobacterium* genomes.

File name: Supplementary Data 3

Description: Counts of sequences across the different LCA levels for the organellar genome mapping analysis. In addition, lists of accession codes are provided for the organellar genomes used as reference.

File name: Supplementary Data 4

Description: Accession codes for the *Nannochloropsis* sequences used in the organellar and *rbc*L, 18S and ITS phylogenies. For the barcode markers the start and end coordinates of the sequence used for the phylogenetic analysis are given.

File name: Supplementary Data 5

Description: The raw linked allele analysis results for both samples and organellar genomes as well as the summarized results.

File name: Supplementary Data 6

Description: The *Nannochloropsis* metabarcode results for the Lake Øvre Æråsvatnet *sed*aDNA and control samples. For each sample, the total number of identified reads, the proportion of the filtered reads identified to the *Nannochloropsis* barcode, and the total number and proportions of PCR replicates containing *Nannochloropsis* is reported.

File name: Supplementary Data 7

Description: The sample names and PCR tags used for the reanalysed Skartjørna, Svalbard (Alsos *et al.* 2016), *sed*aDNA metabarcode dataset, as well as the PCR tags and names for the four

additional metabarcode sequencing libraries from Lake Øvre Æråsvatnet, Andøya, Norway (Alsos *et al.* 2020).

File name: Supplementary Data 8

Description: Detections of *N. limnetica* detections in the re-analysed Lake Hill, St. Paul Island, Alaska, USA (Graham *et al.* 2016; Wang *et al.* 2017), Spring Lake and Charlie Lake, Alberta, Canada (Pedersen *et al.* 2016) and Hässeldala Port, Sweden (Parducci *et al.* 2019) shotgun metagenomic datasets.
