# Supplementary material for: Environmental palaeogenomic reconstruction of an Ice Age algal population
Source: Commun Biol. 2021 Feb 16;4:220. doi: 10.1038/s42003-021-01710-4 (PMC7887274; doi:10.1038/s42003-021-01710-4)
Supplement: Supplementary file 12 — Reporting Summary [file 42003_2021_1710_MOESM12_ESM.pdf]

nature research

Corresponding author(s): Youri Lammers

Last updated by author(s): Dec 11, 2020

Reporting Summary

Nature Research wishes to improve the reproducibility of the work that we publish. This form provides structure for consistency and transparency in reporting. For further information on Nature Research policies, see our Editorial Policies and the Editorial Policy Checklist.

Statistics

For all statistical analyses, confirm that the following items are present in the figure legend, table legend, main text, or Methods section.

n/a	Confirmed	
☒	☐	The exact sample size (*n*) for each experimental group/condition, given as a discrete number and unit of measurement
☒	☐	A statement on whether measurements were taken from distinct samples or whether the same sample was measured repeatedly
☒	☐	The statistical test(s) used AND whether they are one- or two-sided<br>*Only common tests should be described solely by name; describe more complex techniques in the Methods section.*
☒	☐	A description of all covariates tested
☒	☐	A description of any assumptions or corrections, such as tests of normality and adjustment for multiple comparisons
☒	☐	A full description of the statistical parameters including central tendency (e.g. means) or other basic estimates (e.g. regression coefficient) AND variation (e.g. standard deviation) or associated estimates of uncertainty (e.g. confidence intervals)
☒	☐	For null hypothesis testing, the test statistic (e.g. *F*, *t*, *r*) with confidence intervals, effect sizes, degrees of freedom and *P* value noted<br>*Give P values as exact values whenever suitable.*
☒	☐	For Bayesian analysis, information on the choice of priors and Markov chain Monte Carlo settings
☒	☐	For hierarchical and complex designs, identification of the appropriate level for tests and full reporting of outcomes
☒	☐	Estimates of effect sizes (e.g. Cohen's *d*, Pearson's *r*), indicating how they were calculated

Our web collection on statistics for biologists contains articles on many of the points above.

Software and code

Policy information about availability of computer code

Data collection	No software was used for data collection.
Data analysis	SeqPrep v1.2 was used for merging for the paired-end read data.<br>SGA toolkit v0.10.15 was used for the filtering of the merged read data.<br>NCBI-BLAST+ suite v2.2.18+ was used for identification the metagenomic subsets and the re-identification of the mapped genomic data.<br>MEGAN v6.12.3 was used for the visualization of the metagenomic subsets.<br>Bowtie2 v2.3.4.1 was used to map the filtered sequenced onto the reference genomes.<br>SAMtools v0.1.19 was used to parse and filter the mapped sequence data.<br>BCFtools v1.9 was used to call and process the variants.<br>BEDtools v2.17.0 was used to calculate the coverage of the mapped genomes.<br>MEGAHIT pipeline v1.1.4 was used to de novo assemble the metagenomic data.<br>GeSeq v1.77 was used to annotate the reconstructed organellar genomes.<br>OGDRAW v1.3.1 was used to visualize the organellar genomes.<br>mapDamage v2.0.8 was used to inspect the DNA deamination patterns.<br>MAFFT v7.427 was used to produce the organellar and marker alignments.<br>RAxML v8.1.12 was used to produce the organellar and marker phylogenies.<br>PMDtools v0.60 was used to extract deaminated DNA sequences.<br>The script estimating the number of haplotypes across the linked windows is provided in the following GitHub repository at github.com/Y-Lammers/HaplogroupEstimation.

For manuscripts utilizing custom algorithms or software that are central to the research but not yet described in published literature, software must be made available to editors and reviewers. We strongly encourage code deposition in a community repository (e.g. GitHub). See the Nature Research guidelines for submitting code & software for further information.

Data

Policy information about availability of data

All manuscripts must include a data availability statement. This statement should provide the following information, where applicable:

- Accession codes, unique identifiers, or web links for publicly available datasets
- A list of figures that have associated raw data
- A description of any restrictions on data availability

The raw Illumina shotgun sequence datasets, the Lake Øvre Æråsvatnet metabarcode data and the reanalysed metabarcoding data from Alsos et al. 2016 are available via EBML ENA accession: PRJEB38213.
The reconstructed Nannochloropsis limnetica high and low frequency organellar genome sequences are available from NCBI GenBank under accessions MT872223-MT872230.

Field-specific reporting

Please select the one below that is the best fit for your research. If you are not sure, read the appropriate sections before making your selection.

☐ Life sciences ☐ Behavioural & social sciences ☒ Ecological, evolutionary & environmental sciences

For a reference copy of the document with all sections, see nature.com/documents/nr-reporting-summary-flat.pdf

Ecological, evolutionary & environmental sciences study design

All studies must disclose on these points even when the disclosure is negative.

Study description	Two LGM sediment samples from Andoya, northern Norway were shotgun sequenced and metabarcoded. The resulting metagenomic shotgun datasets were analyzed in a number of different ways. First, a subset for both was identified via BLASTing against the NCBI nucleotide database and compared between both sets. Second, the datasets were identified via mapping against a panel of nuclear and organellar reference genomes. Third, the organellar genomes for the algae Nannochloropsis limnetica were reconstructed based on the combined datasets. Fourth, both the organellar genomes (including high and low frequency variants) and the rbcL, ITS and 18S markers were reconstructed for both samples. Fifth, phylogenies were generated for the previous reconstructed organellar genomes and markers and compared between both samples and a set of references. Sixth, the variant sites between the organellar genomes were compared between the samples and the number of present haplotypes were calculated. Seventh, published sedimentary ancient DNA datasets were re-analyzed for the presence of the Nannochloropsis algae. Eighth, the metabarcode data from the same site and lab controls was analysed and checked for the presence of contaminants.
Research sample	The sediment samples were collected from previously collected sediment cores from Andøya, northern Norway.
Sampling strategy	No sample-size collection was performed, instead two sediment samples were selected for shotgun sequencing based on existing metabarcoding taxonomic identifications. Additional samples were selected for further metabarcoding.
Data collection	The two samples were collected and extracted by Inger Greve Alsos in a dedicated ancient DNA laboratory. Library preparation and sequencing was performed by Mikkel Winther Pedersen.
Timing and spatial scale	All samples were sampled and extracted at the same time. Shotgun sequencing and metabarcoding were performed at different times, but utilized the same extracts that were stored at -20 °C.
Data exclusions	No data was excluded.
Reproducibility	Raw data will be made available and can be used to reproduce the results.
Randomization	Randomization is not applicable to this study.
Blinding	Blinding is not applicable to this study.

Did the study involve field work? ☐ Yes ☒ No

Reporting for specific materials, systems and methods

We require information from authors about some types of materials, experimental systems and methods used in many studies. Here, indicate whether each material, system or method listed is relevant to your study. If you are not sure if a list item applies to your research, read the appropriate section before selecting a response.

Materials & experimental systems

n/a	Involved in the study
☒	☐ Antibodies
☒	☐ Eukaryotic cell lines
☒	☐ Palaeontology and archaeology
☒	☐ Animals and other organisms
☒	☐ Human research participants
☒	☐ Clinical data
☒	☐ Dual use research of concern

Methods

n/a	Involved in the study
☒	☐ ChIP-seq
☒	☐ Flow cytometry
☒	☐ MRI-based neuroimaging
